# Fe3O4 nanoparticles and cryoablation enhance ice crystal formation to improve the efficiency of killing breast cancer cells

**DOI:** 10.18632/oncotarget.13859

**Published:** 2016-12-10

**Authors:** Ping Ye, Yu Kong, Xiaojing Chen, Weijie Li, Dejun Liu, Yuexia Xie, Yan Zhou, Hanbing Zou, Zhaohua Chang, Huili Dai, Xianming Kong, Peifeng Liu

**Affiliations:** ^1^ Central Laboratory, Ren Ji Hospital, School of Medicine, Shanghai Jiao Tong University, Shanghai 200127, China; ^2^ State Key Laboratory of Oncogenes and Related Genes, Shanghai Cancer Institute, Ren Ji Hospital, School of Medicine, Shanghai Jiao Tong University, Shanghai 200032, China; ^3^ Shanghai Institute for Minimally Invasive Therapy, School of Medical Instrument and Food Engineering, Shanghai University of Science and Technology, Shanghai 200093, China; ^4^ Shanghai Institutes for Biological Sciences, Chinese Academy of Sciences, Shanghai 200031, China; ^5^ Tumor Hospital, Xinjiang Medical University, Xinjiang 830000, China

**Keywords:** cryoablation, nanoparticles, breast cancer, recrystallization, killing efficiency

## Abstract

The key problem of cryoablation is that only freezing is often unable to kill the capillaries at tumor edges, leading to a high rate of recurrence. Here, we found that Fe_3_O_4_ nanoparticles were highly useful to improve the freezing capability of cryosurgery due to their ability to alter intracellular ice formation (IIF) and growth in tumor cells. The killing efficiency of cryoablation for MCF-7 breast cancer cells can be expected to be enhanced as the Fe_3_O_4_ nanoparticles concentration increased, it was mainly because that more IIF was induced by the participation of Fe_3_O_4_ nanoparticles during freezing, recrystallization and thawing. Furthermore, our results also showed that recrystallization contributed to the formation of extracellular embryonic crystals, which was capable of enhancing the efficiency of killing MCF-7 cells. This research is to develop an understanding of the mechanism of the cryoablation enhancing the killing efficiency in the presence of the Fe_3_O_4_ nanoparticles, and to promote their further application in tumor therapy.

## INTRODUCTION

Cryoablation is a minimally invasive therapy that is commonly used to treat different types of cancer, including breast, liver, lung, prostate, and kidney cancer [1–4]. However, freezing is ineffective at killing the capillaries at tumor edges, leading to a high probability of tumor recurrence [5–9] and greatly impeding the widespread application of cryoablation.

Nanotechnological applications have been widely developed in the medical field, and the nanoparticles involved in cell freezing were recently found to be highly effective in improving the freezing capability of cryosurgery [10–12]. Several attempts have been made to enhance the therapeutic efficiency of cryoablation [13–18], such as the use of gold nanoparticles with high thermal conductivity that could effectively increase the freezing efficiency, amplify the treatment zone of the cryoprobe, and improve the rate of ice ball formation. However, these studies were mainly performed at the macroscopic level, and biological research is needed for further verification. Moreover, the mechanisms for improving the freezing capability of the nanoparticles in cryoablation remain unclear.

It has been demonstrated that intracellular ice formation (IIF) is an important factor influencing the therapeutic efficiency of cryoablation. Additionally, Fe_3_O_4_ nanoparticles could increase the probability of IIF, and effectively improve the killing efficiency of cryoablation for tumor cells during the freeze-thaw process [12, 19, 20]. For this, the present study mainly focused on investigating the mechanism of IIF induced by cryoablation and Fe_3_O_4_ nanoparticles in MCF-7 breast cancer cells and demonstrated that the Fe_3_O_4_ nanoparticles increased the probability of IIF in the freezing process and enhanced recrystallization in the ablation process, which contribute to improve the killing efficiency of tumor cryoablation and reduce the tumor recurrence.

## RESULTS

### Nanoparticle synthesis and characterization

**Figure 1:**
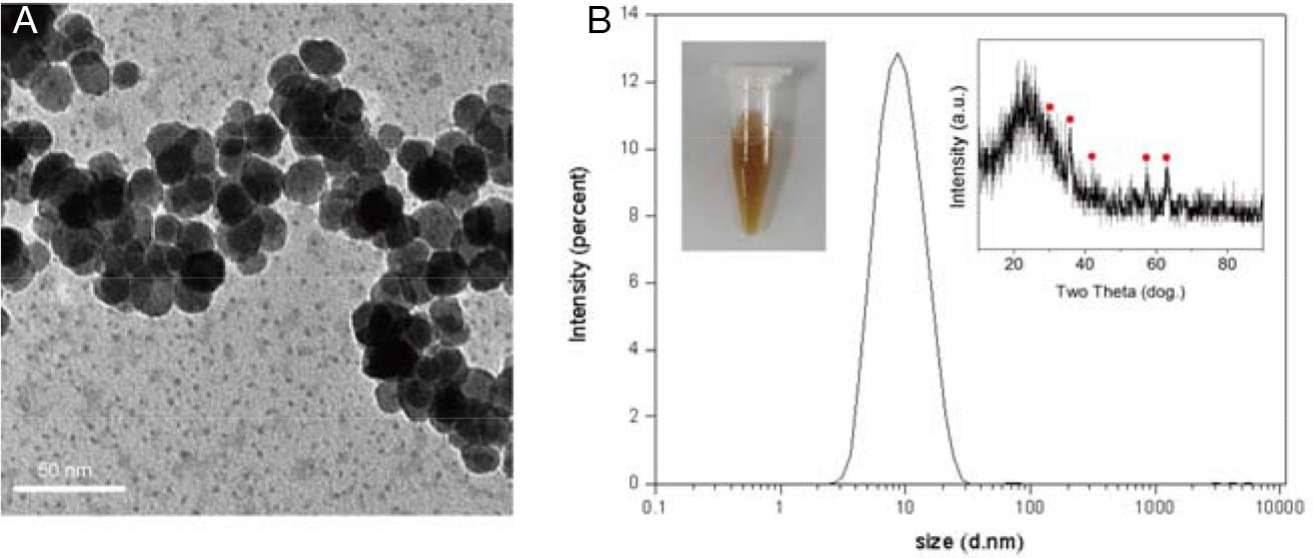
**A**. TEM and **B**. DLS images of the Fe_3_O_4_ nanoparticles. The inserted images (left) and (right) represent Fe_3_O_4_ nanoparticle aqueous solution and XRD image of Fe_3_O_4_ nanoparticle, respectively. The scale bar is 50 nm.

The transmission electron microscope (TEM), X-ray diffraction (XRD) and dynamic light scattering (DLS) results were shown in Figure 1 the Fe_3_O_4_ nanoparticles exhibited a spherical morphology and a diameter of approximately 9 nm with a homogeneous size distribution (Figure 1A and 1B). XRD result confirmed that the observed diffraction pattern could be indexed to Fe_3_O_4_ (JCPDS file 19-0629), demonstrating the Fe_3_O_4_ nanoparticles were successfully synthesized.

### Cytotoxicity

**Figure 2:**
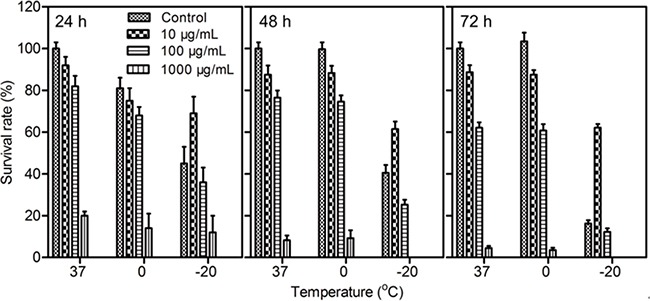
Survival rates of MCF-7 cells treated with Fe_3_O_4_ nanoparticles for 24 h, 48 h or 72 h at 37°C, 0°C or -20°C Data are presented as the mean ± S.D. (n=5).

The effects of cryoablation and Fe_3_O_4_ nanoparticles on the viability of MCF-7 cells are shown in Figure 2. The viabilities of the cells treated with 10 μg/mL and 100 μg/mL Fe_3_O_4_ nanoparticles at 37°C were more than 80%, and the cells exhibited low cytotoxicity compared with the control. In particular, for 1000 μg/mL Fe_3_O_4_ nanoparticles, the cells exhibited the highest cytotoxicity at 37°C, and the survival rate of the cells decreased to 20%, a value that was remarkably lower than that of the cells treated with PBS or 10 μg/mL and 100 μg/mL Fe_3_O_4_ nanoparticles.

With the decrease in cryoablation temperature, the viability of the cells treated with Fe_3_O_4_ nanoparticles became increasingly lower than that of the control. However, the survival rates of cells treated with 10 μg/mL Fe_3_O_4_ nanoparticles at -20°C instead of 37°C or 0°C were higher than that of the control; this finding demonstrated that 10 μg/mL Fe_3_O_4_ nanoparticles enhanced the survival rate of cells in the process of cryoablation, which resulted in a reduction in the killing efficiency for tumor cells, suggesting the concentration of Fe_3_O_4_ nanoparticles is an important factor in the therapeutic efficiency of cryoablation.

### Cell apoptosis

**Figure 3:**
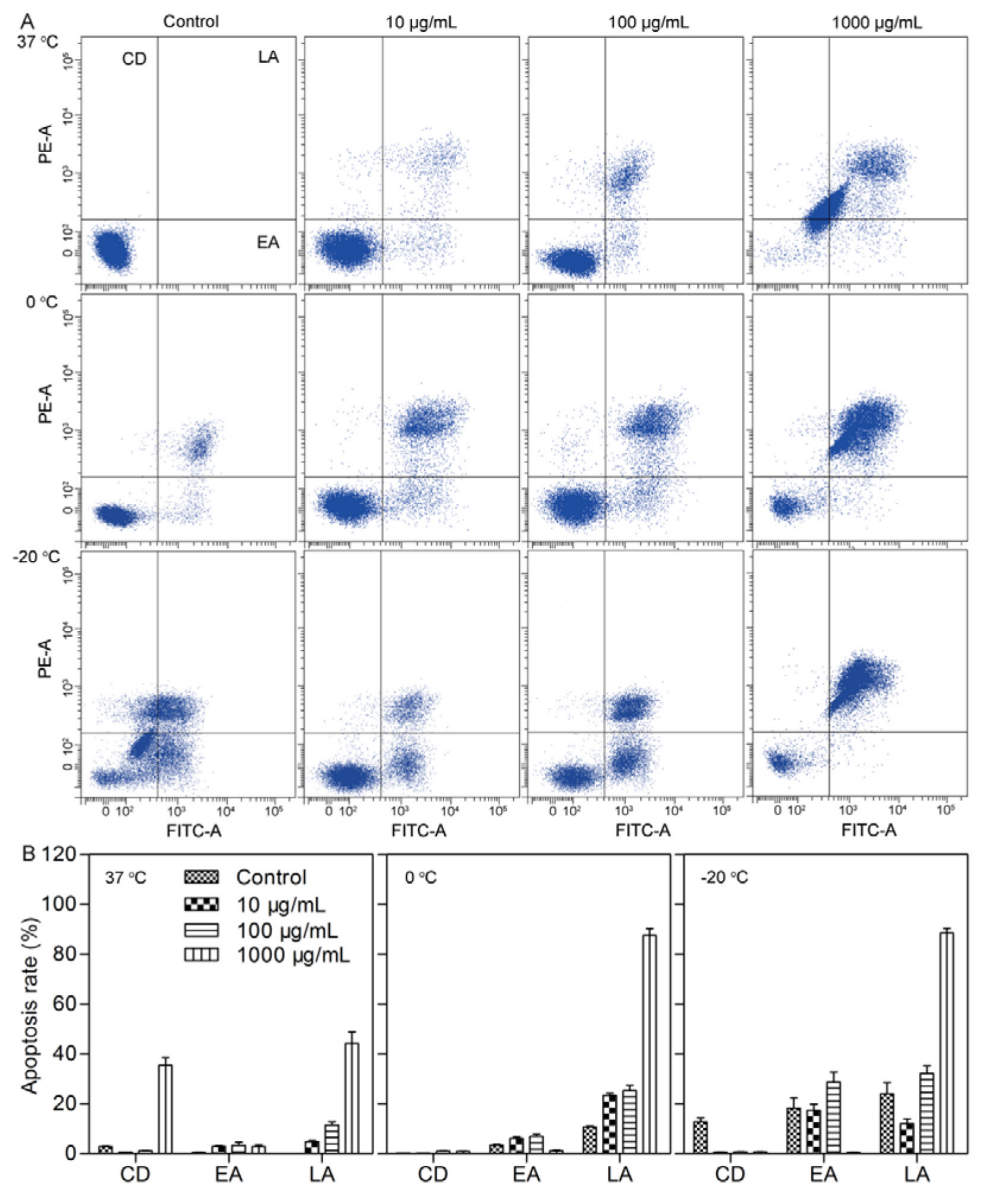
**A**. Flow cytometry analysis of apoptosis in MCF-7 cells treated with Fe_3_O_4_ at 37°C, 0°C and -20°C for 72 h. **B**. Quantitative data of the apoptosis in MCF-7 cells treated with Fe_3_O_4_ at 37°C, 0°C and -20°C for 72 h. CD, EA and LA represent cell debris, early apoptosis and late apoptosis, respectively. Data are presented as the mean ± S.D. (n=3).

Cell apoptosis was measured to evaluate the mechanism underlying the cytotoxicity induced by cryoablation and the Fe_3_O_4_ nanoparticles [21]. As shown in Figure 3, the cell debris (CD) and late apoptosis (LA) of MCF-7 cells treated with 1000 μg/mL Fe_3_O_4_ nanoparticles were obviously higher than those of other groups at 37°C, demonstrating that the CD and LA of cells were the main reasons of the high cytotoxicity induced by a high concentration Fe_3_O_4_ nanoparticles.

Because the cell viability subsequently reduced as the temperature decreased, the CD of the MCF-7 cells at 0°C and -20°C also decreased compared with that at 37°C. In contrast, the early apoptosis (EA) and LA constantly increased as the temperature decreased from 0°C to -20°C, suggesting that the low temperature influenced the viability of cells through EA and LA. However, the addition of the Fe_3_O_4_ nanoparticles led to a different result. Specifically, 10 μg/mL Fe_3_O_4_ nanoparticles induced a decrease in EA and LA at -20°C compared with that of the control; in contrast, 100 μg/mL Fe_3_O_4_ nanoparticles induced an increase in EA and LA, and 1000 μg/mL Fe_3_O_4_ nanoparticles also induced an increase in LA. These results explained the increased cell viability caused by 10 μg/mL Fe_3_O_4_ nanoparticles at -20°C; however, compared with the control, 100 μg/mL and 1000 μg/mL Fe_3_O_4_ nanoparticles caused a decrease in cell viability at -20 oC, which was mainly induced by enhancing the LA.

### Cryomicroscopy observation of IIF

**Figure 4:**
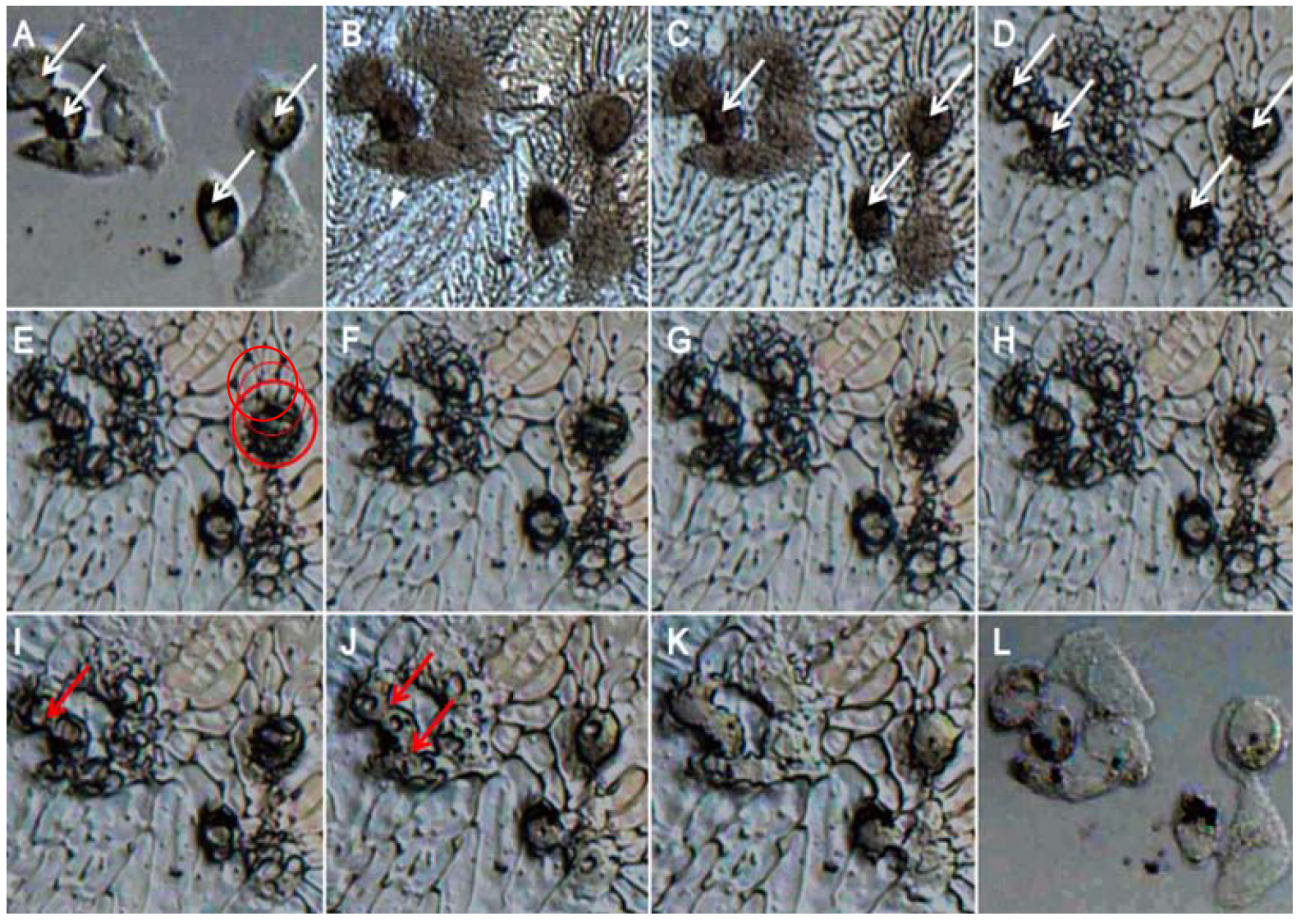
Cell cryomicroscopy images of MCF-7 cells during the freezing and thawing process MCF-7 cells were treated with 100 μg/mL Fe_3_O_4_ nanoparticles and were washed with PBS twice before freezing. **A**. At 20°C before freezing. **B**. At -13.6°C during freezing. **C**. At -13.8°C during freezing. **D**. At -16.3°C during freezing. **E**. At -25.9°C during freezing. **F**. At -32.6°C during freezing. **G**. At -40°C, the final state before thawing. **H**. At -30.7°C during thawing. **I**. At -17.7°C during thawing. **J**. At -11.3°C during thawing. **K**. At -7.9°C during thawing. **L**. At 20°C after thawing.

Cryomicroscopy was employed to monitor the variation of IIF in MCF-7 cells treated with cryoablation and Fe_3_O_4_ nanoparticles. As shown in Figure 4, Fe_3_O_4_ nanoparticles were phagocytized into cells after incubation for 3 h (labeled by white arrows) (Figure 4A). During the freezing process, small extracellular ice crystals were formed, and these crystals covered the MCF-7 cells when the temperature decreased to -13.6°C; the white triangles indicated the direction of the extracellular ice growth (Figure 4B).

Subsequently, intracellular ice gradually formed in the cytoplasm at -13.8°C (labeled by white arrows) (Figure 4C) and then rapidly gathered and formed larger crystals (labeled by white arrows) (Figure 4D) when the temperature n cell viability at -20 oC, which was mainly induced by enhancing the LA. Decreased to -16.3°C. As the temperature continued to decrease to -25.9°C, a few intracellular ice crystals penetrated the nuclear membrane but did not cover the entire nucleus (labeled by a red circle) (Figure 4E). The formation rate of the intracellular ice gradually decreased with the temperature change from -25.9°C to -40°C, especially for the temperature range between -30°C and -40°C. Additionally, the intracellular ice almost stopped growing when cooling or thawing occurred (Figure 4F, )G and H), largely due to the decrease in supercooling degree.

During the thawing process, as the temperature increased from -40°C to -17.7°C, embryonic crystals entered the cells to be recrystallized in the cytoplasm, and small intracellular ice crystals first formed large crystals (labeled by red arrows) (Figure 4I and 4J), which contributed to enhance the killing efficiency for tumor cells. As the temperature continued to increase, the large crystals gradually melted (Figure 4J) and finally melted completely at -7.9°C (Figure 4K). After thawing, the cell borders were blurred to obscurity, suggesting that the cytomembrane was damaged (Figure 4L).

### The killing efficiency of recrystallization

**Figure 5:**
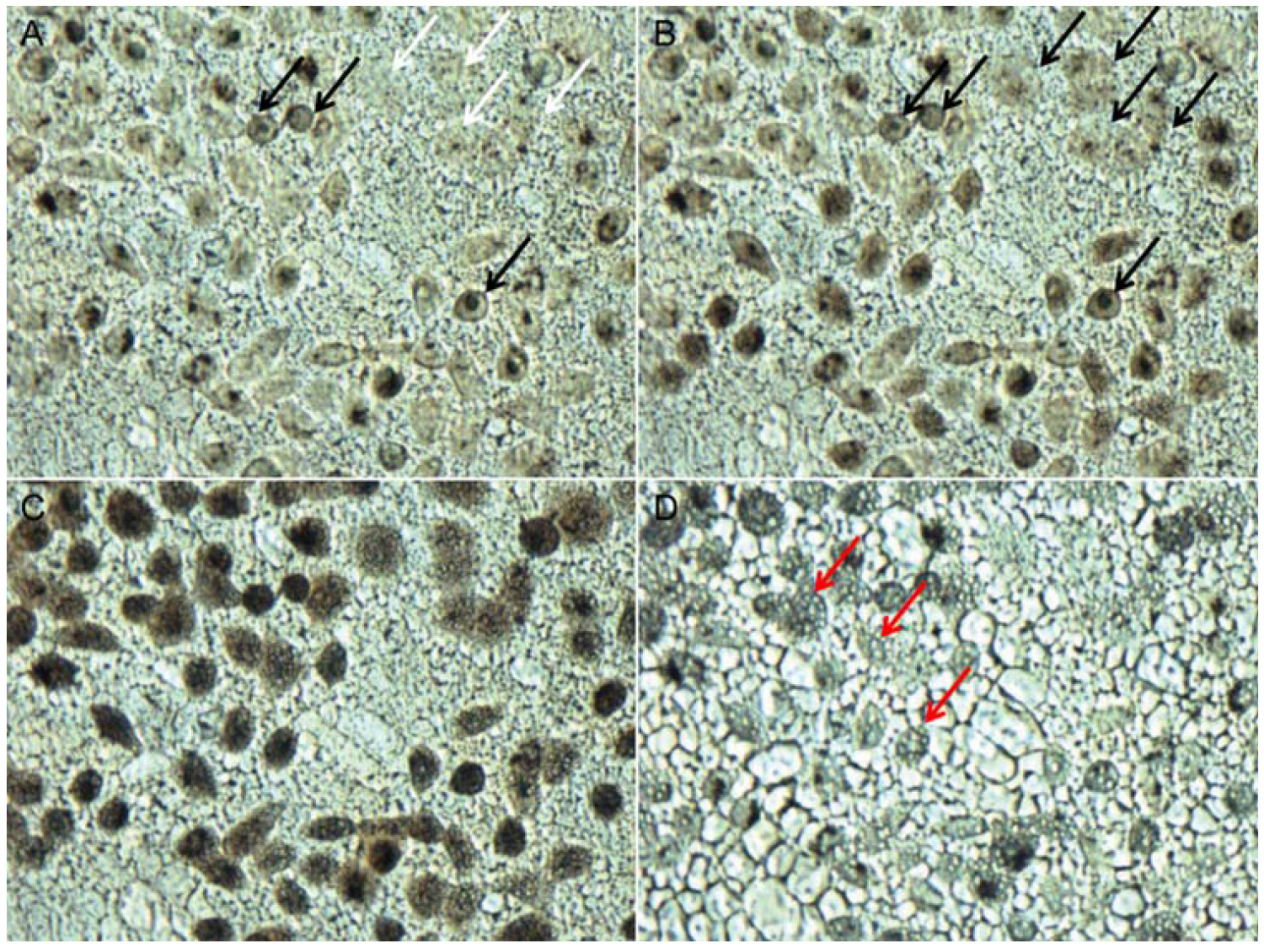
Recrystallization killing of MCF-7 cells during the ablation process MCF-7 cells were treated with 100 μg/mL MgNPs-Fe_3_O_4_ and were warmed at a heating rate of 100°C/min from -40°C to room temperature. **A**. Image before recrystallization at -21°C. **B**. Image during recrystallization at -19°C. **C**. Image during recrystallization at -13.5°C. **D**. Image after recrystallization at -7°C. (Scale bar is 20 μm).

Since cryoablation is capable of leading to the recrystallization of IIF, therefore, we carried out the experiment to evaluate the killing efficiency of recrystallization on tumor cells. As shown in Figure 5A, the black arrows indicated intracellular ice, and the white arrows indicated cells without intracellular ice. The MCF-7 cells with intracellular ice were gray and translucent before recrystallization. As the temperature increased from -21°C to -19°C, the intracellular ice began to recrystallize, and it gradually grew in the cytoplasm and penetrated the nucleus, making the nucleus display a black color. (Figure 5B). When the intracellular ice gradually grew and covered the entire intracellular cytoplasmic and nuclear space, MCF-7 cells became completely black and opaque (Figure 5C). As the temperature increased to -7°C, the intracellular ice in the cytoplasm and nucleus melted and formed small holes (labeled by red arrows) (Figure 5D), demonstrating the effective killing induced by recrystallization in MCF-7 cells.

### IIF change in cryoablation

**Figure 6:**
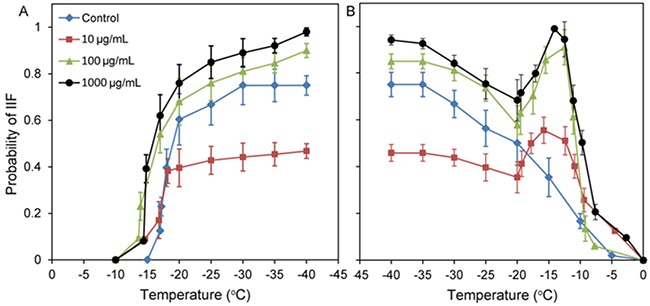
**A**. Probability of IIF at a freezing rate of 5°C/min in MCF-7 cells treated with 0 (control), 10 μg/mL, 100 μg/mL and 1000 μg/mL Fe_3_O_4_ nanoparticles for 3 h. **B**. Probability of IIF at a heating rate of 100°C/min in MCF-7 cells treated with 0 (control), 10 μg/mL, 100 μg/mL or 1000 μg/mL Fe_3_O_4_ nanoparticles for 3 h. Data are presented as the mean ± S.D. (n=5).

The freezing process is shown in Figure 6A, the temperature of IIF in the control is -17°C, and the probability of IIF gradually increased as the freezing temperature decreased. However, with the addition of 10 μg/mL, 100 μg/mL and 1000 μg/mL Fe_3_O_4_ nanoparticles, the temperature of IIF clearly changed and increased to -13.6°C. This change resulted from the higher thermal conductivity of Fe_3_O_4_ nanoparticles compared with tumor and healthy tissue, which led to the increased freezing rate and enhanced probability of IIF, thus contributing to freeze and kill tumor cells.

The probability of IIF in MCF-7 cells treated with 10 μg/mL Fe_3_O_4_ nanoparticles in the -20°C to -40°C temperature range was obviously lower than that of the cells treated with the control and with the 100 μg/mL and 1000 μg/mL Fe_3_O_4_ nanoparticles, demonstrating that the IIF probability was closely associated with the concentration of Fe_3_O_4_ nanoparticles.

The thawing process is shown in Figure 6B; the probability of IIF in cells decreased as the temperature increased. No recrystallization phenomenon was observed in the control. However, in MCF-7 cells treated with 10 μg/mL, 100 μg/mL and 1000 μg/mL Fe_3_O_4_ nanoparticles, a recrystallization phenomenon was clearly observed at -20°C. As the temperature increased from -20°C to -15°C, the probability of IIF also increased accordingly; this finding was completely different from the thawing process for the control, demonstrating an effective influence of Fe_3_O_4_ nanoparticles for recrystallization during the thawing process.

### TEM observation of cryoablation

**Figure 7:**
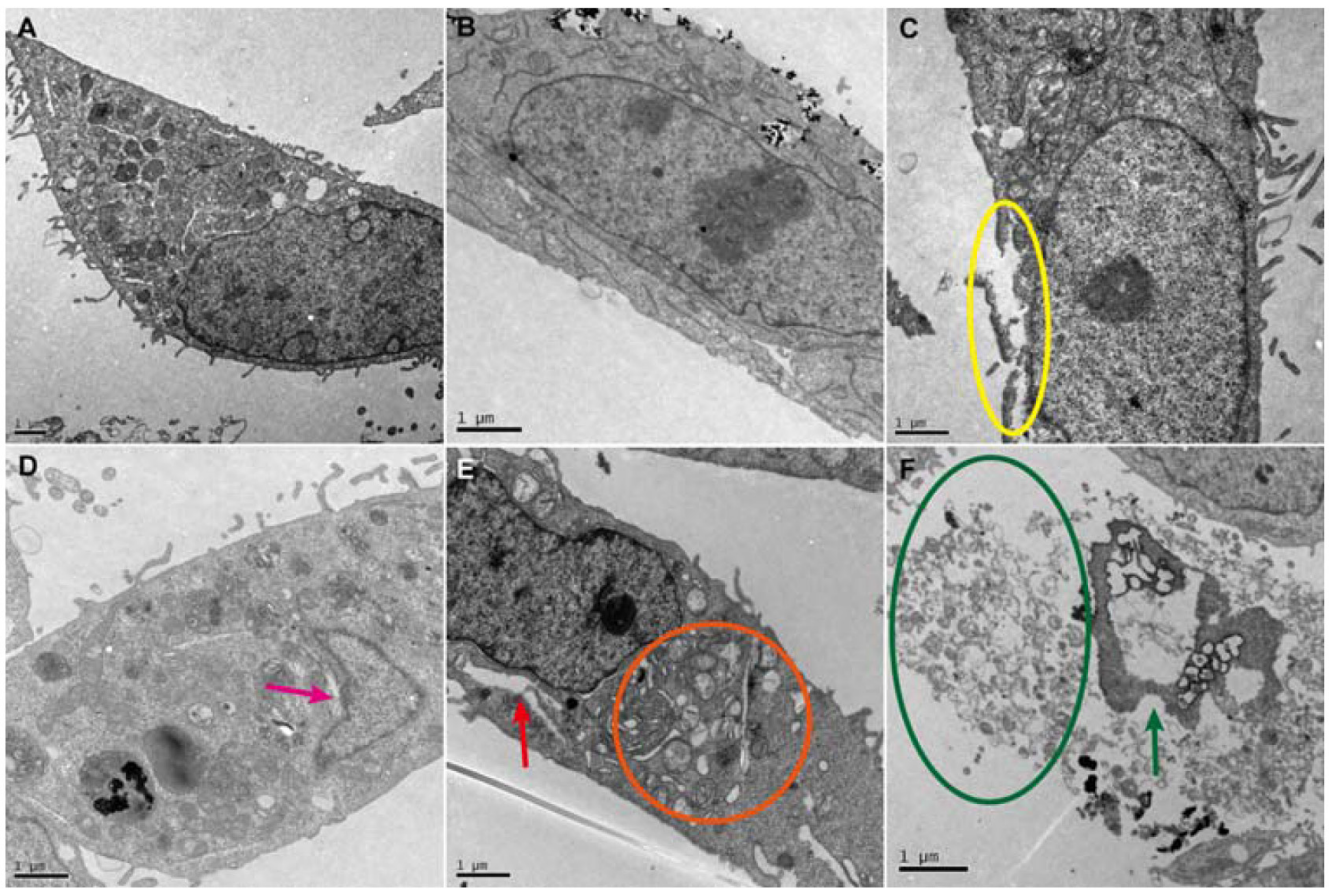
TEM images of MCF-7 cells treated with cryoablation and Fe_3_O_4_ nanoparticles **A**. Control. **B**. Cellular uptake of 1000 μg/mL Fe_3_O_4_ nanoparticles. **C**. Cryoablation. **D**. Cryoablation + 10 μg/mL Fe_3_O_4_ nanoparticles. **E**. Cryoablation + 100 μg/mL Fe_3_O_4_ nanoparticles. **F**. Cryoablation + 1000 μg/mL Fe_3_O_4_ nanoparticles.

TEM was used to observe the ultrastructure variation of cells treated with Fe_3_O_4_ nanoparticles [22–24]. As shown in Figure 7A, the cells in the control group were smooth and intact. A number of mitochondria with a uniform size and high electron density were observed in the cytoplasm. Additionally, the organelle structure was clear, and the cells grew well. Subsequently, the cellular uptake of Fe_3_O_4_ nanoparticles was evaluated, and the results showed that Fe_3_O_4_ nanoparticles (1000 μg/mL) were effectively internalized by cells and mainly accumulated in the vesicles. Although the cell membrane structure was intact, cells presented mild edema and ridge swelling, and the mitochondria of the cells also increased, suggesting that the high concentration Fe_3_O_4_ nanoparticles caused cell damage (Figure 7B). After the cells were treated via cryoablation, the cell membrane was severely damaged by the ice crystal growth after cryoablation (labeled by a yellow circle), the mitochondria became swollen, and there were broken ridges (Figure 7C).

With the addition of 10 μg/mL Fe_3_O_4_ nanoparticles, we found that the cell membrane exhibited a complete structure; the effects from the ice crystals formed by cryoablation were less than that for the treatment with cryoablation alone (labeled by a pink arrow) (Figure 7D). This observation can be attributed to the inhibition of ice crystal formation in the presence of 10 μg/mL Fe_3_O_4_ nanoparticles during cryoablation, resulting in a lower efficiency of killing tumor cells. However, when the concentration of Fe_3_O_4_ nanoparticles increased to 100 μg/mL, the cells were greatly damaged by cryoablation, and deformed nuclei, empty mitochondria, decreased or lost ridges and different-sized vesicles in the rough surface of the endoplasmic reticulum were clearly observed (labeled by an orange circle). Furthermore, an obvious cavity structure caused by cryoablation was found in the cytoplasm (labeled by a red arrow) (Figure 7E). As the concentration of Fe_3_O_4_ nanoparticles increased to 1000 μg/mL, the damage by cryoablation for cells was most severe; the profile of the cells disappeared and was completely disintegrated into fragments (labeled by a green circle). Only nuclear fragments and few or no organelles remained (labeled by a green arrow) (Figure 7F), demonstrating the efficient killing of tumor cells.

## DISCUSSION

In this study, we carried out experiments to investigate the validity of Fe_3_O_4_ nanoparticles in improving the freezing capability of cryosurgery in tumor cells and discussed the relative mechanisms. The results suggested that 10 μg/mL Fe_3_O_4_ nanoparticles was more effective to enhance the survival rate of cells in the process of cryoablation as compared with 100 μg/mL and 1000 μg/mL Fe_3_O_4_ nanoparticles, demonstrating that the concentration of Fe_3_O_4_ nanoparticles exhibited a differentiated influence on the therapeutic efficiency of cryoablation. Similar results were also observed in the analysis of cell apoptosis. Subsequently, we simulated the entire process of cryoablation in the presence of 100 μg/mL Fe_3_O_4_ nanoparticles, confirming that the ice crystals were efficiently formed during the freezing and thawing process, which was considered to perform twice killing for MCF-7 breast cancer cells [25].

A major obstacle in cryoablation is the ineffectiveness in killing the cells at the tumor edges. For cryoablation, IIF is a key factor in the process of killing tumor cells [19]. We found that 100 μg/mL and 1000 μg/mL Fe_3_O_4_ nanoparticles concentration-dependently increased the probability of IIF. However, 10 μg/mL nanoparticles decreased the probability of IIF as compared with the control, presumably due to the reason that Fe_3_O_4_ nanoparticles with the concentration of 10 μg/mL generated a protective effect on the viability of MCF-7 cells at -20°C as compared with the viabilities for 100 μg/mL and 1000 μg/mL Fe_3_O_4_ nanoparticles.

Recrystallization is a key factor that induces direct injury in tumor cells during the thawing process of cryoablation [20]. In our study, the heating rate of 100°C/min was used to simulate the rapid ablation process of cryoablation [13], and in the clinical setting, rapid ablation contributes to a reduction in complications, pain and recovery time [12]. We found that IIF only appeared through recrystallization in the presence of Fe_3_O_4_ nanoparticles when the temperature increased from -20°C to -15°C. The IIF probability gradually increased as the Fe_3_O_4_ nanoparticle concentration increased, allowing the killing of tumor cells by recrystallization during the thawing process.

TEM observation demonstrated that the killing of MCF-7 breast cancer cells by cryoablation gradually improved as the concentration of Fe_3_O_4_ nanoparticles increased from 10 μg/mL to 1000 μg/mL, presumably due to the reason that Fe_3_O_4_ nanoparticles tended to gather in nanoparticle clusters when the concentration of Fe_3_O_4_ nanoparticles increased, leading to an increase in the volume and surface area of nanoparticles and a reduction of interfacial energy. This result facilitated the formation of embryo crystallites from H_2_O molecules on the surface of clusters through cryoablation, resulting in the high probability of IIF during the freezing process of cryoablation and the formation of large ice crystals fused from small ice crystals during the thawing process of cryoablation [24, 26]. Thus, the efficiency of killing tumor cells was significantly improved. Our work demonstrated the underlying mechanism of the enhanced killing efficiency of Fe_3_O_4_ nanoparticles in cryoablation, and therefore, provided theoretical foundation for the application of Fe_3_O_4_ nanoparticles in cryoablation.

## MATERIALS AND METHODS

### Materials

Fe(acac)_3_ was purchased from Acros Organics USA (Pittsburgh, PA, USA). Fetal bovine serum (FBS) and Dulbecco's modified Eagle medium (DMEM) were purchased from Thermo Fisher Scientific (Waltham, MA, USA). CCK-8 reagent was purchased from Dojindo Chemical Technology (Shanghai) Co., Ltd. (Shanghai, China). An Annexin V-FITC/PI apoptosis kit was purchased from Sigma-Aldrich China Co., Ltd. (Shanghai, China). Other reagents were purchased from Sinopharm Chemical Reagent Co., Ltd. (Shanghai, China).

The MCF-7 cell line was obtained from the Shanghai Cell Institute Country Cell Bank and was grown in high-glucose DMEM (Gibco, USA) containing 10% fetal bovine serum (Gibco, USA), 100 U/mL penicillin and 100 μg/mL streptomycin (Hyclone, USA) at 37°C in a 5% CO_2_ atmosphere with 95% relative humidity.

### Nanoparticle synthesis and characterization

Nine-nanometer Fe_3_O_4_ nanoparticles were prepared using the polyol method. Briefly, 2 mmol of Fe(acac)_3_ and 25 mL of triethylene glycol were directly added to a three-neck round-bottomed flask equipped with a condenser, magnetic stirrer, thermograph, and heating mantle; then, the mixture was stirred under argon. The mixture was heated to 180°C at a rate of 3°C min^-1^ and was kept at that temperature for 30 min, followed by quick heating to reflux (-280°C) and maintaining reflux for another 30 min. After cooling down to room temperature, obtaining a black homogeneous colloidal suspension containing magnetite nanoparticles. The homogeneous colloidal suspension was dialyzed in distilled water and then was collected with a magnet to obtain Fe_3_O_4_ magnetic nanoparticles. Subsequently, 2 mmol of the Fe(acac)_3_ was again added to the solution to react according to the above condition. Finally, the solution was dialyzed in distilled water and collected to obtain 9 nm Fe_3_O_4_ magnetic nanoparticles.

The morphology and size distribution of the Fe_3_O_4_ nanoparticles were separately characterized using an HT7700 transmission electron microscope (TEM) (Hitachi, Japan) and Mestersizer 2000 dynamic light scattering (DLS) analyzer (Malvern, UK), respectively.

### Cryoablation device

**Figure 8:**
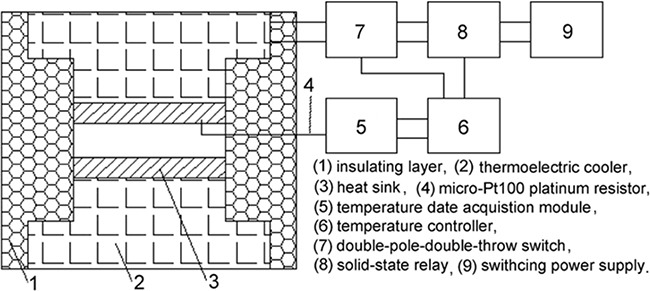
Diagram of the structure of the temperature-control device used for the cryoablation of cells

To maintain consistent experimental conditions during the heating-cooling cycles of the MCF-7 cells undergoing cryoablation, we designed a temperature-controlled cell culture device consisting of a temperature-controlled box and a temperature-control system (Figure 8). The temperature-controlled box comprises an insulating layer, a semiconductor chilling plate and a copper conductor. The temperature-control system comprises a multi-stage semiconductor thermoelectric cooler that was used to homogenize and stabilize the temperature. The temperature range of the device can be regulated in the range of -60 to 60°C. The cooling rate can be controlled from 0.1 to 10°C/min, and the heating rate can be controlled from 0.1 to 200°C/min.

### Cytotoxicity

The MTT assay was used to evaluate the killing efficiency of cryoablation and Fe_3_O_4_ nanoparticles for MCF-7 cells. Briefly, MCF-7 cells (3 × 10^3^ cells/well) were seeded in 96-well plates and incubated for 24 h. Next, 10 μg/mL, 100 μg/mL and 1000 μg/mL Fe_3_O_4_ nanoparticles were added and incubated for 3 h. The cells were then subjected to 37°C, 0°C and -20°C for 15 min, followed by incubation for 24 h, 48 h, 72 h, 96 h or 120 h. Next, 20 μL of MTT (5 mg/mL) was added, and the cells were incubated for an additional 4 h. The culture medium was replaced with 200 μL of DMSO. Finally, the absorbance was measured at a wavelength of 490 nm using a Bio-Rad 680 microplate reader (Bio-Rad Laboratories, California, USA).

### Cell apoptosis

The apoptosis level of MCF-7 cells was determined using a FACScan flow cytometer. Briefly, MCF-7 cells (1 × 10^5^ cells/well) were seeded in 6-well plates and were incubated for 24 h. Next, 10 μg/mL, 100 μg/mL and 1000 μg/mL Fe_3_O_4_ nanoparticles were separately added and incubated for 3 h. The cells were then subjected to 37°C, 0°C and -20°C for 15 min, followed by incubation for 72 h. The cells were then washed with PBS, trypsinized, harvested, and resuspended in 500 μL of binding buffer. Finally, 2 μL of Annexin V-FITC and 5 μL of propidium iodide were added, and the cells were incubated for 5 min. The apoptosis level of the MCF-7 cells was measured using a FACScan flow cytometer from Becton Dickinson (New York, USA), which was operated at an excitation wavelength of 488 nm and an emission wavelength of 530 nm.

### Cryomicroscopy

To evaluate the influence of cryoablation and Fe_3_O_4_ nanoparticles on MCF-7 cells, we constructed a cryomicroscopy system consisting of a Linkam BCS 196 cryostage (Linkam Scientific Instrument, UK), a microscope (LSM 510 META; Carl Zeiss Microscopy, Germany), a TMS94 temperature controller (Linkam Scientific Instrument, UK), a high-speed CMOS camera (the Cooke Corporation, USA) and Linksys 32-DV control software (Linkam Scientific Instrument, UK). The temperature range of the cryostage could be regulated from -196 to 200°C, and the heating-cooling rate could be controlled from 0.01 to 100/min.

MCF-7 cells (2.5 × 10^4^ cells/well) were seeded on the cover glasses in 24-well plates and were incubated for 24 h. Next, 10 μg/mL, 100 μg/mL and 1000 μg/mL Fe_3_O_4_ nanoparticles were separately added, and the cells were incubated for 3 h. The culture medium was removed, and then the cells were covered with another cover glass. Subsequently, the cover glass with cells was placed on the cryostage. The cells were cooled from room temperature to -40°C at 5°C/min for 1 min to achieve equilibrium, and then they were warmed to room temperature at a speed of 100°C/min. The freezing and ablation process was imaged using a cryomicroscopy with a long working distance objective of 50 magnification, and the data were recorded by a digital high speed CMOS camera system (Cooke Corporation, USA). The exposure time in the camera could range from 50 ns to 5 s. The camera system was controlled by the Windows application Camware (the Cooke Corporation, USA).

### IIF probability of cryoablation

To confirm the mechanism of cryoablation, we quantified the variation in the IIF probability during cryoablation. Briefly, MCF-7 cells (2.5 × 10^4^ cells/well) were seeded on cover glasses in 24-well plates and were incubated for 24 h. Fe_3_O_4_ nanoparticles with the concentrations of 10 μg/mL, 100 μg/mL and 1000 μg/mL were added, and the cells were incubated for 3 h. The culture medium was removed, a drop of culture medium was added to the cover glass with cells, and then the cells were covered with another cover glass. Subsequently, the cover glass with cells was placed on the cryostage. The cells were cooled from room temperature to -40°C at 5°C/min for 1 min to achieve equilibrium; finally, they were warmed to room temperature at a speed of 100°C/min. The entire freezing and ablation processes were recorded using cryomicroscopy. The total number of MCF-7 cells in the cell image before freezing was determined. The number of MCF-7 cells in the image that had experienced IIF during freezing and ablation was determined. The probability of IIF as a function of temperature is shown below:

$$\text{Probability of IIF}=\frac{The\;number\;of\;cells\;that\;has\;undergone\;IIF\;at\;a\;certain\;temperature}{Total\;number\;of\;cells\;in\;the\;observation}$$

### Killing efficiency of recrystallization

A recrystallization experiment was performed to confirm the killing efficiency for tumor cells. Briefly, MCF-7 cells (2.5 × 10^4^ cells/well) were seeded on cover glasses in 24-well plates and were incubated for 24 h. Next, 10 μg/mL, 100 μg/mL and 1000 μg/mL Fe_3_O_4_ nanoparticles were added, and then the cells were incubated for 3 h. The culture medium was removed, a drop of culture medium was added to the cover glass with cells, and then the cells were covered with another cover glass. Subsequently, the cover glass with cells was placed on the cryostage. The cells were cooled from room temperature to -40°C at a speed of 5°C/min for 1 min to achieve equilibrium. Finally, the cells were warmed to room temperature at a speed of 100°C/min. The entire process was observed using cryomicroscopy.

### TEM observation of cryoablation

TEM was used to evaluate the influence of cryoablation on the ultrastructural alterations of MCF-7 cells. Briefly, MCF-7 cells (1 × 10^5^ cells/well) were seeded in 6-well plates and were incubated for 24 h. Next, 10 μg/mL, 100 μg/mL and 1000 μg/mL Fe_3_O_4_ nanoparticles were separately added to each well, and then the cells were incubated for 3 h. The cells were then subjected to 37°C and -20°C for 15 min each, followed by immediate thawing to 37°C and incubation for another 2 h. Next, the cells were washed with 0.1 M PBS, embedded in a 2% agarose gel, post fixed in 4% osmium tetroxide solution for 1 h, stained with 0.5% uranyl acetate for 1 h, dehydrated in a graded series of ethanol (30%, 60%, 70%, 90% and 100%), and embedded in epoxy resin. The resin was polymerized at 60°C for 48 h. Ultrathin sections obtained with a ultramicrotome were stained with 5% aqueous uranyl acetate and 2% aqueous lead citrate and air dried, then imaged using TEM (JEOL JEM2100, Japan).
